# *Research Communications* – over 100 papers and counting!

**DOI:** 10.1107/S1600536814023393

**Published:** 2014-10-31

**Authors:** Helen Stoeckli-Evans

**Affiliations:** a Institute of Physics, University of Neuchâtel, Rue Emile-Argand 11, CH-2000 Neuchâtel, Switzerland

**Keywords:** crystallography, editorial, Acta E

The relaunch of *Acta Crystallographica Section E* in June heralded its transformation from *Structure Reports Online* to *Crystallographic Communications* and saw the publication of the first papers in the new *Research Communication* format designed to bring out the science behind the structure determination. Reports are no longer limited to a short description of a single structure and figures are no longer relegated to the Supporting information. We are delighted to see that an increasing number of authors are choosing to publish their work as a *Research Communication*, with the numbers of these articles increasing every month. The November issue includes the one hundredth paper to be published in this format, so what better time to celebrate the great breadth in the range and scope of the *Research Communications* published so far?

Authors are positively encouraged to report and discuss related structures in a single *Research Communication* rather than publishing a series of short structural reports. We are pleased to see that papers reporting two structures are a now regular feature and one three-structure paper has been published. Authors have made the most of the opportunity to include extra tables and figures in the published paper to illustrate their results and enhance the discussion of the underlying science.

We are pleased to see that the variety of compounds covered is vast, and includes a fullerene, a potential secondary explosive, a herbicide, MOF’s, a two-dimensional grid-type structure, a layered coordination polymer, a four-layered [3.3](3,5)pyridino­phane, an anti-HIV agent, a new solid form of the drug seratrodast, a helicene, isotypic structures, polymorphs and caged compounds to name but a few. While the majority of crystals have been measured with Mo *K*α or Cu *K*α radiation, some studies have benefited from synchrotron and even neutron time-of-flight measurements.

These full papers have discussed some very interesting science, the findings of the studies including, amongst others, an example of the importance of freely refining the positions of the amino-group H atoms, the influence of solvent upon the stoichiometry of the formed salt, relative substituent orientation, extension of the coordination chemistry of a certain ligand type farther towards the early transition metals, and the influence of the molecular shape on the hydrogen-bonding pattern. 

Each month a Section Editor has the pleasure of choosing the structure for the cover. The cover choices reflect the broad range of topics you will find among the *Research Communications*. In July, the cover image was taken from the first *Research Communication* ever to be published, which reports alkyne dicobalt clusters featuring asymmetric or symmetric alkynes, classic tetrahedral C_2_Co_2_ cluster cores, with each carbon atom of the alkyne bridging two Co atoms, and highly distorted octahedral geometries. A two-dimensional grid-type structure with the organic guest molecules occupying the space between adjacent grid layers was the choice in August, whereas the molecular structure of 2,7-diethoxy-1,8-bis(4-nitrobenzoyl)naphthalene, which possesses crystallographically imposed twofold symmetry and whose crystal packing is characterized by C—H⋯O=C and C—H⋯O=N hydrogen bonds appeared in September. The October cover featured two molybdenum(II) cyclopentadienyl complexes in which the steric demands of the different phosphine ligands have a minor effect on bond lengths and angles within the complex molecule whereas the crystal packing in the two structures is markedly different, with the acetyl oxygen atom playing a crucial role as an acceptor of non-classical C—H⋯O hydrogen bonds. This month, the choice relates to the interesting chemistry and crystallography of *N*-aryl­hydroxylamines, where four independent mol­ecules are linked *via* O—H⋯N and N—H⋯N hydrogen bonds to form a tetramer-like unit. 

We would like to see even more of our authors old and new submit to the journal in this format. Our editors and many of our authors already take advantage of the IUCr’s *publCIF* software to write and edit their papers using a word-processing environment. *publCIF* takes a crystallographic information file (CIF) and prepares a formatted paper (preprint) in the *Research Communication* style. The CIF and the preprint are presented side-by-side and can both be edited – changes made to one are applied to the other as you type. *publCIF* also includes many useful editorial tools to help you write your paper. It can be used to add data items required for publication, prepare standard and customized geometry tables, check your CIF for both syntax and completeness, print or export a preprint of your paper, check the references and more. Using *publCIF*, a *Research Communication* can be produced with just a little extra effort. Be sure to use the latest version of *publCIF*, which is available to download free of charge from http://publcif.iucr.org.

A large part of the success of the new *Research Communication* format is down to our dedicated team of Co-editors who are doing an excellent job in advising authors how to promote their science. On behalf of the Section Editors of *Acta E*, I would like to take this opportunity to thank them for helping make *Acta Crystallographica Section E* the obvious choice for disseminating the results of the excellent crystallography that is being carried out by our authors worldwide.

